# 2-[2-(2-Hy­droxy­eth­oxy)phen­yl]-4,4,5,5-tetra­methyl-2-imidazoline-1-oxyl 3-oxide

**DOI:** 10.1107/S1600536811050860

**Published:** 2011-11-30

**Authors:** Lin-Lin Jing, Hui-Ping Ma, Lei He, Peng-Cheng Fan, Zheng-Ping Jia

**Affiliations:** a Department of Pharmacy, Lanzhou General Hospital of PLA, Key Laboratory of the Prevention and Cure for the Plateau Environment Damage, PLA 730050, Lanzhou Gansu, People’s Republic of China

## Abstract

In the title compound, C_15_H_21_N_2_O_4_, the nitronyl nitroxide unit displays a twisted conformation. The crystal structure is stabilized by non-classical C—H⋯O and C—H⋯π hydrogen bonds, which build up a three-dimensional network.

## Related literature

For the biological activity of nitronyl nitroxides, see: Soule *et al.* (2007 ▶); Blasig *et al.* (2002 ▶); Qin *et al.* (2009 ▶); Tanaka *et al.* (2007 ▶). For their coordination properties, see: Masuda *et al.* (2009 ▶). For puckering parameters, see: Cremer & Pople (1975 ▶). For pseudorotation parameters, see: Rao *et al.* (1981 ▶).
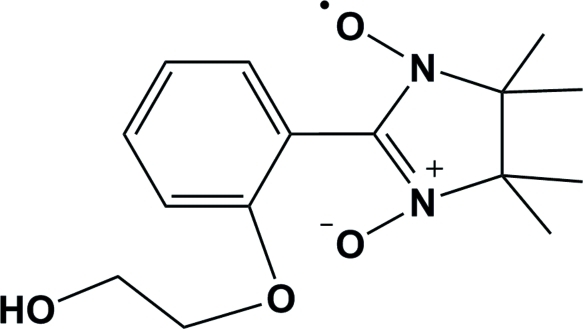


## Experimental

### 

#### Crystal data

C_15_H_21_N_2_O_4_
*M* *_r_* = 293.34
Orthorhombic, 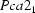

*a* = 14.458 (7) Å
*b* = 10.187 (5) Å
*c* = 10.670 (5) Å
*V* = 1571.5 (13) Å^3^
*Z* = 4
Mo *K*α radiation
μ = 0.09 mm^−1^
*T* = 296 K
0.23 × 0.20 × 0.19 mm

#### Data collection

Bruker APEXII CCD diffractometer
Absorption correction: multi-scan (*SADABS*; Bruker, 2007 ▶) *T*_min_ = 0.980, *T*_max_ = 0.983
10740 measured reflections
1547 independent reflections
1245 reflections with *I* > 2σ(*I*)
*R*_int_ = 0.038

#### Refinement

*R*[*F* ^2^ > 2σ(*F* ^2^)] = 0.042
*wR*(*F* ^2^) = 0.107
*S* = 1.05
1547 reflections
195 parameters
1 restraint
H-atom parameters constrained
Δρ_max_ = 0.11 e Å^−3^
Δρ_min_ = −0.14 e Å^−3^

### 

Data collection: *APEX2* (Bruker, 2007 ▶); cell refinement: *SAINT* (Bruker, 2007 ▶); data reduction: *SAINT*; program(s) used to solve structure: *SHELXS97* (Sheldrick, 2008 ▶); program(s) used to refine structure: *SHELXL97* (Sheldrick, 2008 ▶); molecular graphics: *ORTEP-3* (Farrugia, 1997 ▶); software used to prepare material for publication: *SHELXTL* (Sheldrick, 2008 ▶) and *PLATON* (Spek, 2009 ▶).

## Supplementary Material

Crystal structure: contains datablock(s) I, global. DOI: 10.1107/S1600536811050860/rk2315sup1.cif

Structure factors: contains datablock(s) I. DOI: 10.1107/S1600536811050860/rk2315Isup2.hkl

Additional supplementary materials:  crystallographic information; 3D view; checkCIF report

## Figures and Tables

**Table 1:** Hydrogen-bond geometry (Å, °) *Cg*2 is the centroid of the C4–C9 ring.

*D*—H⋯*A*	*D*—H	H⋯*A*	*D*⋯*A*	*D*—H⋯*A*
C8—H8⋯O1^i^	0.93	2.45	3.248 (5)	143
C6—H6⋯O1^ii^	0.93	2.50	3.364 (5)	155
C14—H14*B*⋯*Cg*2^iii^	0.96	3.00	3.513 (5)	115

Symmetry codes: (i) 
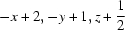
; (ii) 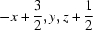; (iii) 
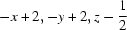
.

**Fig. 1.**
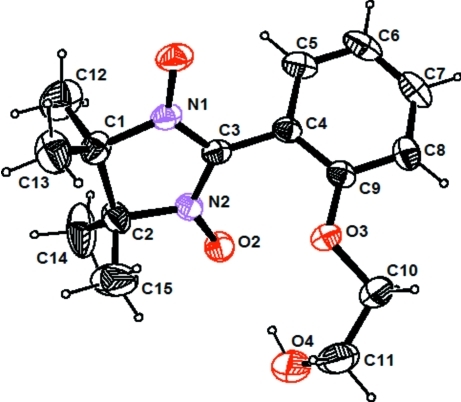
Molecular structure of the title compound with the atom numbering scheme. Displacement ellipsoids are drawn at the 30% probability level. H atoms are presented as a small spheres of arbitrary radius.

## supplementary crystallographic information

### Comment

Nitronyl nitroxides, firstly synthesized more than 30 years ago, can be used for coordination with many metalcations, such as Mn^2+^, Cu^2+^ and Ni^2+^ leading to form some molecule based magentic materials (Masuda *et al.*, 2009). They also can react with free radicals such as OH, H_2_O_2_, and O_2_ (Blasig *et al.*, 2002) to protect cells from the attack of free radicals. So they have lots of biological properities as anticancer, antiradiation and antioxidation (Qin *et al.*, 2009; Tanaka *et al.*, 2007; Soule *et al.*, 2007).

The molecular structure of the title compound is shown in Fig. 1. The nitronyl nitroxide ring and the phenyl rings are twisted with respect to each other making a dihedral angle of 50.07 (9)°. The puckering parameters of the nitronyl nitroxide ring are Q(2) = 0.143 (4)Å and φ = 236.4 (14)° (Cremer & Pople, 1975). The pseudorotation parameters (Rao *et al.*, 1981) for the nitronyl nitroxide ring are P = 39.5 (9)° and τ(*M*) = 14.8 (2)° for the C1—N1 reference bond with the closest puckering descriptor being twisted on C1—C2.

The crystal structure is stabilized by non–classical intermolecular C—H···O and C—H···π hydrogen bonds (Table 1).

### Experimental

2,3–Dimethyl–2,3–bis(hydroxylamino) butane (1.48 g, 10.0 mmol) and 2–(2–hydroxyethoxy)benzaldehyde (1.66 g, 10 mmol) were dissolved in methanol (30.0 ml). The reaction was filtered after stirring for 24 h at room temperature. The resulting white powder was washed by cool methanol and suspended in the solution of dichloromethane (30.0 ml). Then the reaction mixture was added to an aqueous solution of NaIO_4_(30 ml) and stirred for 15 min in an ice bath to give a dark red solution. The aqueous phase was extracted with CH_2_Cl_2_ and the organic layer was combined and dried over Na_2_SO_4_. Then the solvent was removed to give a dark red residue which was purified by flash column chromatography with the elution of *n*–hexane / ethyl acetate (1:3) to yield 1.69 g (57%) of the title compound as a dark red powder. Single crystals of the title compound suitable for X–ray diffraction was recrystallized from hexane / dichloromethane (2:1).

### Refinement

In the structure all the H atoms were positioned geometrically and refined with using a riding model: C—H_methyl_ = 0.96Å; C—H_methylene_ = 0.97Å; C—H_aryl_ = 0.93Å and O—H = 0.82Å with *U*_iso_(H) = 1.2*U*_eq_(C), *U*_iso_(H) = 1.5*U*_eq_(C_methyl_) and *U*_iso_(H) = 1.5*U*_eq_(O).

### Crystal data

**Table:**

C_15_H_21_N_2_O_4_	*F*(000) = 628
*M**_r_* = 293.34	*D*_x_ = 1.240 Mg m^−^^3^
Orthorhombic, *P**c**a*2_1_	Mo *K*α radiation, λ = 0.71073 Å
Hall symbol: P 2c -2ac	Cell parameters from 2696 reflections
*a* = 14.458 (7) Å	θ = 2.8–23.3°
*b* = 10.187 (5) Å	µ = 0.09 mm^−^^1^
*c* = 10.670 (5) Å	*T* = 296 K
*V* = 1571.5 (13) Å^3^	Block, red
*Z* = 4	0.23 × 0.20 × 0.19 mm

### Data collection

**Table:**

Bruker APEXII CCD diffractometer	1547 independent reflections
Radiation source: fine–focus sealed tube	1245 reflections with *I* > 2σ(*I*)
graphite	*R*_int_ = 0.038
φ and ω scans	θ_max_ = 25.5°, θ_min_ = 2.5°
Absorption correction: multi-scan (*SADABS*; Bruker, 2007)	*h* = −17→17
*T*_min_ = 0.980, *T*_max_ = 0.983	*k* = −11→12
10740 measured reflections	*l* = −12→12

### Refinement

**Table:**

Refinement on *F*^2^	Primary atom site location: structure-invariant direct methods
Least-squares matrix: full	Secondary atom site location: difference Fourier map
*R*[*F*^2^ > 2σ(*F*^2^)] = 0.042	Hydrogen site location: inferred from neighbouring sites
*wR*(*F*^2^) = 0.107	H-atom parameters constrained
*S* = 1.05	*w* = 1/[σ^2^(*F*_o_^2^) + (0.0518*P*)^2^ + 0.287*P*] where *P* = (*F*_o_^2^ + 2*F*_c_^2^)/3
1547 reflections	(Δ/σ)_max_ < 0.001
195 parameters	Δρ_max_ = 0.11 e Å^−^^3^
1 restraint	Δρ_min_ = −0.14 e Å^−^^3^

### Special details

**Table:**

Geometry. All s.u.'s (except the s.u. in the dihedral angle between two l.s. planes) are estimated using the full covariance matrix. The cell s.u.'s are taken into account individually in the estimation of s.u.'s in distances, angles and torsion angles; correlations between s.u.'s in cell parameters are only used when they are defined by crystal symmetry. An approximate (isotropic) treatment of cell s.u.'s is used for estimating s.u.'s involving l.s. planes.
Refinement. Refinement of *F*^2^ against ALL reflections. The weighted *R*–factor *wR* and goodness of fit *S* are based on *F*^2^, conventional *R*–factors *R* are based on *F*, with *F* set to zero for negative *F*^2^. The threshold expression of *F*^2^ > σ(*F*^2^) is used only for calculating *R*–factors(gt) *etc*. and is not relevant to the choice of reflections for refinement. *R*–factors based on *F*^2^ are statistically about twice as large as those based on *F*, and *R*–factors based on ALL data will be even larger.

### Fractional atomic coordinates and isotropic or equivalent isotropic displacement parameters (Å^2^)

**Table:**

	*x*	*y*	*z*	*U*_iso_*/*U*_eq_	
C1	0.9017 (2)	0.8466 (3)	0.2894 (4)	0.0611 (10)	
C2	0.9954 (3)	0.9121 (4)	0.3217 (4)	0.0651 (10)	
C3	0.95885 (19)	0.7595 (3)	0.4802 (3)	0.0464 (8)	
C4	0.9624 (2)	0.6861 (3)	0.5974 (3)	0.0461 (7)	
C5	0.8832 (3)	0.6738 (4)	0.6705 (4)	0.0636 (10)	
H5	0.8274	0.7071	0.6410	0.076*	
C6	0.8859 (3)	0.6144 (4)	0.7837 (5)	0.0804 (14)	
H6	0.8327	0.6074	0.8321	0.097*	
C7	0.9683 (3)	0.5645 (4)	0.8264 (5)	0.0793 (12)	
H7	0.9705	0.5253	0.9050	0.095*	
C8	1.0473 (3)	0.5710 (3)	0.7558 (4)	0.0632 (10)	
H8	1.1023	0.5356	0.7858	0.076*	
C9	1.0443 (2)	0.6313 (3)	0.6386 (3)	0.0469 (8)	
C10	1.2080 (2)	0.6121 (4)	0.6047 (5)	0.0749 (11)	
H10A	1.2186	0.5184	0.6130	0.090*	
H10B	1.2165	0.6529	0.6860	0.090*	
C11	1.2719 (3)	0.6687 (6)	0.5129 (5)	0.0959 (16)	
H11A	1.3346	0.6439	0.5348	0.115*	
H11B	1.2584	0.6322	0.4309	0.115*	
C12	0.8185 (3)	0.9359 (4)	0.3095 (8)	0.115 (2)	
H12A	0.7632	0.8839	0.3127	0.173*	
H12B	0.8143	0.9973	0.2415	0.173*	
H12C	0.8257	0.9828	0.3869	0.173*	
C13	0.8961 (4)	0.7819 (5)	0.1625 (4)	0.0905 (15)	
H13A	0.9469	0.7220	0.1529	0.136*	
H13B	0.8991	0.8477	0.0983	0.136*	
H13C	0.8389	0.7347	0.1557	0.136*	
C14	0.9919 (5)	1.0609 (4)	0.3315 (6)	0.134 (3)	
H14A	0.9462	1.0858	0.3924	0.201*	
H14B	0.9759	1.0975	0.2515	0.201*	
H14C	1.0513	1.0934	0.3570	0.201*	
C15	1.0746 (3)	0.8629 (9)	0.2416 (5)	0.139 (3)	
H15A	1.1323	0.8875	0.2794	0.209*	
H15B	1.0704	0.9009	0.1595	0.209*	
H15C	1.0713	0.7690	0.2352	0.209*	
N1	0.89542 (17)	0.7436 (3)	0.3891 (3)	0.0524 (7)	
N2	1.01428 (18)	0.8600 (2)	0.4494 (3)	0.0505 (7)	
O1	0.83122 (15)	0.6579 (2)	0.3892 (3)	0.0682 (7)	
O2	1.07936 (16)	0.9064 (2)	0.5172 (2)	0.0612 (6)	
O3	1.11657 (14)	0.6369 (2)	0.5582 (2)	0.0522 (6)	
O4	1.2663 (2)	0.8039 (4)	0.5064 (5)	0.1191 (15)	
H4	1.2119	0.8265	0.5099	0.179*	

### Atomic displacement parameters (Å^2^)

**Table:**

	*U*^11^	*U*^22^	*U*^33^	*U*^12^	*U*^13^	*U*^23^
C1	0.063 (2)	0.0458 (19)	0.074 (3)	0.0010 (16)	−0.0135 (19)	0.0018 (18)
C2	0.077 (3)	0.064 (2)	0.054 (2)	−0.0238 (19)	−0.009 (2)	0.0130 (19)
C3	0.0374 (15)	0.0421 (17)	0.060 (2)	−0.0002 (13)	0.0027 (15)	−0.0036 (14)
C4	0.0460 (16)	0.0375 (16)	0.0550 (19)	−0.0034 (13)	0.0071 (15)	−0.0021 (15)
C5	0.056 (2)	0.056 (2)	0.079 (3)	−0.0072 (17)	0.0181 (19)	−0.004 (2)
C6	0.088 (3)	0.069 (3)	0.085 (3)	−0.017 (2)	0.042 (3)	0.006 (2)
C7	0.109 (3)	0.063 (2)	0.066 (2)	−0.015 (2)	0.021 (3)	0.017 (2)
C8	0.076 (2)	0.048 (2)	0.065 (2)	−0.0004 (18)	−0.001 (2)	0.0116 (18)
C9	0.0517 (18)	0.0333 (16)	0.056 (2)	−0.0035 (13)	0.0059 (16)	−0.0031 (15)
C10	0.051 (2)	0.081 (3)	0.093 (3)	0.0064 (19)	−0.005 (2)	0.010 (3)
C11	0.052 (2)	0.135 (5)	0.101 (4)	−0.002 (3)	0.002 (3)	−0.001 (3)
C12	0.091 (3)	0.071 (3)	0.184 (6)	0.027 (2)	−0.010 (4)	0.018 (4)
C13	0.118 (4)	0.084 (3)	0.070 (3)	−0.029 (3)	−0.022 (3)	0.003 (2)
C14	0.210 (6)	0.069 (3)	0.123 (4)	−0.059 (4)	−0.085 (5)	0.045 (3)
C15	0.074 (3)	0.285 (9)	0.059 (3)	−0.037 (4)	0.007 (2)	−0.004 (4)
N1	0.0412 (14)	0.0455 (15)	0.0705 (18)	−0.0027 (12)	−0.0021 (14)	−0.0004 (14)
N2	0.0514 (15)	0.0435 (14)	0.0565 (16)	−0.0070 (12)	−0.0023 (14)	0.0029 (13)
O1	0.0477 (12)	0.0592 (15)	0.0977 (19)	−0.0149 (11)	−0.0061 (14)	−0.0009 (14)
O2	0.0633 (14)	0.0583 (14)	0.0620 (14)	−0.0200 (12)	−0.0142 (13)	0.0013 (12)
O3	0.0411 (11)	0.0581 (13)	0.0573 (15)	0.0037 (10)	0.0004 (10)	−0.0029 (11)
O4	0.0635 (18)	0.130 (3)	0.164 (4)	−0.026 (2)	−0.006 (2)	0.056 (3)

### Geometric parameters (Å, °)

**Table:**

C1—N1	1.498 (5)	C10—C11	1.464 (6)
C1—C13	1.508 (6)	C10—H10A	0.9700
C1—C12	1.522 (5)	C10—H10B	0.9700
C1—C2	1.550 (5)	C11—O4	1.381 (6)
C2—N2	1.488 (5)	C11—H11A	0.9700
C2—C15	1.514 (7)	C11—H11B	0.9700
C2—C14	1.520 (6)	C12—H12A	0.9600
C3—N2	1.341 (4)	C12—H12B	0.9600
C3—N1	1.346 (4)	C12—H12C	0.9600
C3—C4	1.458 (5)	C13—H13A	0.9600
C4—C9	1.381 (4)	C13—H13B	0.9600
C4—C5	1.391 (5)	C13—H13C	0.9600
C5—C6	1.352 (6)	C14—H14A	0.9600
C5—H5	0.9300	C14—H14B	0.9600
C6—C7	1.372 (6)	C14—H14C	0.9600
C6—H6	0.9300	C15—H15A	0.9600
C7—C8	1.369 (6)	C15—H15B	0.9600
C7—H7	0.9300	C15—H15C	0.9600
C8—C9	1.394 (5)	N1—O1	1.274 (3)
C8—H8	0.9300	N2—O2	1.277 (3)
C9—O3	1.353 (4)	O4—H4	0.8200
C10—O3	1.435 (4)		
			
N1—C1—C13	109.1 (3)	O4—C11—C10	112.9 (4)
N1—C1—C12	105.7 (4)	O4—C11—H11A	109.0
C13—C1—C12	110.2 (4)	C10—C11—H11A	109.0
N1—C1—C2	101.3 (3)	O4—C11—H11B	109.0
C13—C1—C2	115.8 (4)	C10—C11—H11B	109.0
C12—C1—C2	113.7 (3)	H11A—C11—H11B	107.8
N2—C2—C15	105.1 (4)	C1—C12—H12A	109.5
N2—C2—C14	107.4 (3)	C1—C12—H12B	109.5
C15—C2—C14	113.3 (5)	H12A—C12—H12B	109.5
N2—C2—C1	102.1 (3)	C1—C12—H12C	109.5
C15—C2—C1	113.2 (4)	H12A—C12—H12C	109.5
C14—C2—C1	114.6 (4)	H12B—C12—H12C	109.5
N2—C3—N1	108.8 (3)	C1—C13—H13A	109.5
N2—C3—C4	125.5 (3)	C1—C13—H13B	109.5
N1—C3—C4	125.6 (3)	H13A—C13—H13B	109.5
C9—C4—C5	119.4 (3)	C1—C13—H13C	109.5
C9—C4—C3	120.7 (3)	H13A—C13—H13C	109.5
C5—C4—C3	119.9 (3)	H13B—C13—H13C	109.5
C6—C5—C4	121.1 (4)	C2—C14—H14A	109.5
C6—C5—H5	119.4	C2—C14—H14B	109.5
C4—C5—H5	119.4	H14A—C14—H14B	109.5
C5—C6—C7	119.2 (4)	C2—C14—H14C	109.5
C5—C6—H6	120.4	H14A—C14—H14C	109.5
C7—C6—H6	120.4	H14B—C14—H14C	109.5
C8—C7—C6	121.5 (4)	C2—C15—H15A	109.5
C8—C7—H7	119.2	C2—C15—H15B	109.5
C6—C7—H7	119.2	H15A—C15—H15B	109.5
C7—C8—C9	119.3 (4)	C2—C15—H15C	109.5
C7—C8—H8	120.4	H15A—C15—H15C	109.5
C9—C8—H8	120.4	H15B—C15—H15C	109.5
O3—C9—C4	116.3 (3)	O1—N1—C3	125.3 (3)
O3—C9—C8	124.3 (3)	O1—N1—C1	121.7 (3)
C4—C9—C8	119.3 (3)	C3—N1—C1	112.8 (3)
O3—C10—C11	106.3 (3)	O2—N2—C3	125.7 (3)
O3—C10—H10A	110.5	O2—N2—C2	121.4 (3)
C11—C10—H10A	110.5	C3—N2—C2	112.8 (3)
O3—C10—H10B	110.5	C9—O3—C10	119.0 (3)
C11—C10—H10B	110.5	C11—O4—H4	109.5
H10A—C10—H10B	108.7		
			
N1—C1—C2—N2	−13.7 (3)	O3—C10—C11—O4	66.3 (5)
C13—C1—C2—N2	−131.6 (3)	N2—C3—N1—O1	−179.0 (3)
C12—C1—C2—N2	99.3 (4)	C4—C3—N1—O1	−3.2 (5)
N1—C1—C2—C15	98.7 (4)	N2—C3—N1—C1	−4.4 (4)
C13—C1—C2—C15	−19.2 (5)	C4—C3—N1—C1	171.4 (3)
C12—C1—C2—C15	−148.3 (5)	C13—C1—N1—O1	−50.7 (4)
N1—C1—C2—C14	−129.4 (4)	C12—C1—N1—O1	67.9 (4)
C13—C1—C2—C14	112.7 (5)	C2—C1—N1—O1	−173.3 (3)
C12—C1—C2—C14	−16.5 (6)	C13—C1—N1—C3	134.5 (3)
N2—C3—C4—C9	−52.9 (4)	C12—C1—N1—C3	−107.0 (4)
N1—C3—C4—C9	132.0 (3)	C2—C1—N1—C3	11.9 (4)
N2—C3—C4—C5	125.4 (3)	N1—C3—N2—O2	176.5 (3)
N1—C3—C4—C5	−49.7 (4)	C4—C3—N2—O2	0.7 (5)
C9—C4—C5—C6	3.1 (5)	N1—C3—N2—C2	−5.9 (4)
C3—C4—C5—C6	−175.3 (3)	C4—C3—N2—C2	178.3 (3)
C4—C5—C6—C7	−0.6 (6)	C15—C2—N2—O2	72.3 (4)
C5—C6—C7—C8	−1.4 (7)	C14—C2—N2—O2	−48.5 (5)
C6—C7—C8—C9	0.9 (6)	C1—C2—N2—O2	−169.4 (3)
C5—C4—C9—O3	174.4 (3)	C15—C2—N2—C3	−105.4 (4)
C3—C4—C9—O3	−7.2 (4)	C14—C2—N2—C3	133.8 (4)
C5—C4—C9—C8	−3.6 (5)	C1—C2—N2—C3	12.9 (4)
C3—C4—C9—C8	174.8 (3)	C4—C9—O3—C10	164.8 (3)
C7—C8—C9—O3	−176.2 (3)	C8—C9—O3—C10	−17.3 (5)
C7—C8—C9—C4	1.7 (5)	C11—C10—O3—C9	−160.6 (3)

### Hydrogen-bond geometry (Å, °)

**Table:**

Cg2 is the centroid of the C4–C9 ring.

**Table:**

*D*—H···*A*	*D*—H	H···*A*	*D*···*A*	*D*—H···*A*
C8—H8···O1^i^	0.93	2.45	3.248 (5)	143
C6—H6···O1^ii^	0.93	2.50	3.364 (5)	155
C14—H14B···Cg2^iii^	0.96	3.00	3.513 (5)	115

Symmetry codes: (i) −*x*+2, −*y*+1, *z*+1/2; (ii) −*x*+3/2, *y*, *z*+1/2; (iii) −*x*+2, −*y*+2, *z*−1/2.
